# Effects of "Bioactive" amino acids leucine, glutamate, arginine and tryptophan on feed intake and mRNA expression of relative neuropeptides in broiler chicks

**DOI:** 10.1186/2049-1891-3-27

**Published:** 2012-08-24

**Authors:** Songbo Wang, Paul Khondowe, Shengfeng Chen, Jianjian Yu, Gang Shu, Xiaotong Zhu, Lina Wang, Ping Gao, Qianyun Xi, Yongliang Zhang, Qingyan Jiang

**Affiliations:** 1 College of Animal Sciences, South China Agricultural University, Guangzhou, 510642, P. R. China; 2 School of Natural Sciences, Department of Biological Sciences, University of Zambia, P.O. Box 32379, Lusaka, Zambia

**Keywords:** Broiler chicks, Feed intake, Hypothalamus, Intracerebroventricular (ICV), L-leucine, L-glutamate

## Abstract

Feed intake control is vital to ensuring optimal nutrition and achieving full potential for growth and development in poultry. The aim of the present study was to investigate the effects of L-leucine, L-glutamate, L-tryptophan and L-arginine on feed intake and the mRNA expression levels of hypothalamic Neuropeptide involved in feed intake regulation in broiler chicks. Leucine, glutamate, tryptophan or arginine was intra-cerebroventricularly (ICV) administrated to 4d-old broiler chicks respectively and the feed intake were recorded at various time points. Quantitative PCR was performed to determine the hypothalamic mRNA expression levels of Neuropeptide Y (NPY), agouti related protein (AgRP), pro-opiomelanocortin (POMC), melanocortin receptor 4 (MC4R) and corticotrophin releasing factor (CRF). Our results showed that ICV administration of L-leucine (0.15 or 1.5  μmol) significantly (*P* < 0.05) increased feed intake up to 2 h post-administration period and elevated both hypothalamic NPY and AgRP mRNA expression levels. In contrast, ICV administration of L-glutamate (1.6  μmol) significantly (*P* < 0.05) decreased feed intake 0.25, 0.5 and 2 h post-injection, and increased hypothalamic CRF and MC4R mRNA expression levels. Meanwhile, both L-tryptophan (10 or 100  μg) and L-arginine (20 or 200  μg) had no significant effect on feed intake. These findings suggested that L-leucine and L-glutamate could act within the hypothalamus to influence food intake, and that both orexigenic and anorexigenic Neuropeptide genes might contribute directly to these effects.

## Background

Metabolic fuels, including amino acids, could act on hypothalamic neurons to regulate feeding behavior and energy homeostasis, but the signaling mechanisms mediating these effects are not fully clear [1]. The arcuate nucleus (ARC) of the hypothalamus contains at least two distinct groups of neurons controlling feeding behavior and energy balance, which are, neurons that contain the orexigenic Neuropeptide (including NPY and AgRP) and neurons that contain the anorexigenic Neuropeptide (including POMC) [2,3]; and from the ARC, neurons project to ‘second order neurons’ in the paraventricular nucleus (PVN) (responsible for producing corticotrophin-releasing factor (CRF), a potent anorexigenic peptide), ventromedial hypothalamus area (VMH), and lateral hypothalamic area (LHA) to orchestrate feeding behavior [3,4].

In mammals, recent data indicated that brain amino acid sensing also contributed to the homeostatic regulation of food intake and body weight [5]. In avian, many of the classic neurotransmitters, including amino acids, had been shown to affect food intake when injected directly into the central nervous system [6,7].

Leucine, glutamate, tryptophan and arginine are among the important ‘bioactive amino acids’, and participate in many important and diverse biochemical reactions associated with the normal physiology of the organism. Leucine, an essential amino acid, likely represents a physiological signal of hypothalamic amino acid availability [8]. It enters the brain more quickly than other amino acids, and it is the most potent activator of the amino-acid-sensitive mTORC1 pathway in mammals [8,9]. Leucine intracerebroventricular (ICV) injection led to a decrease in food intake in mammals, by increasing hypothalamic mTOR signaling [5]. In addition, It was reported that providing a diet deficient in valine but with excess leucine resulted in a rapid decrease in feed intake in pigs [10]. In contrast, central administration of leucine significantly stimulated food intake in neonatal chicks [11]. However, the effect of ICV injection of leucine on hypothalamic orexigenic and anorexigenic Neuropeptide remained to be elucidated.

L-glutamate is the most abundant free amino acid in brain and is the predominant excitatory neurotransmitter of the vertebrate central nervous system. Glutamate was previously demonstrated to be an endogenous agent involved in the neural control of food intake and body weight in mammals [12,13]. Systemic, ICV or local administration of glutamate or glutamate agonists into the lateral hypothalamus could evoke a dose-related stimulation of food intake in mammals [12,14]. Nonetheless, currently there is still little information on the impact of glutamate on feeding behavior in chickens, and results have contrasted those obtained in mammals [15-17].

Tryptophan, an essential amino acid, is the precursor for the synthesis of serotonin. In mammals, the effect of tryptophan deficiency on growth was mainly associated with a reduction of appetite and feed intake [18,19]. For poultry, dietary tryptophan significantly elevated body weight gain and feed intake [20], while central administration had been shown to suppress food intake in free fed chicks [21]. There is, however, limited research information on the effects of central administration of tryptophan on hypothalamic orexigenic and anorexigenic Neuropeptide in broiler chicks.

L-arginine is one of the metabolically versatile amino acids, giving rise to nitric oxide (NO); and NO is recognized as one of feeding-regulatory factors in the brain of mammals [22]. In mammals, central administration of L-arginine with leptin blocked leptin’s inhibitory effects on food intake and NOS activity [23,24]. In chickens, L-arginine ICV injection had been shown to attenuate the decrease in food intake induced by leptin [25]. However, there is currently limited research information on the effects of L-arginine central administration on feed intake in broiler chickens.

Therefore, the purpose of the present study was to determine the effects of ICV administration of the amino acids leucine, glutamate, tryptophan, and arginine on feed intake and to elucidate the contribution of hypothalamic orexigenic and anorexigenic Neuropeptide to the effects of these amino acids in broiler chicks. We measured the feed intake at various time points after central injection. Additionally, the hypothalamic mRNA expression levels of NPY, AgRP, POMC, MC4R, and CRF were tested.

## Materials and methods

### Experimental animals

1-d old broiler chicks were purchased from South China Agricultural University Hatchery and were maintained in a room at a constant temperature of 30 ± 1°C. Lighting was provided continuously for 24 h every day. Chicks were given free access to a commercial starter diet and water. 1 d prior to the experimental day, the chicks (3-d old) were selected and distributed into 12 experimental groups based on their body weight and average feed intake, so that the average body weight was as uniform as possible within the same experimental group. All experimental procedures followed the guidance for animal experiments and handling of the College of Animal Science of South China Agricultural University.

### Preparation of drugs

The amino acids L-leucine, L-glutamate, L-arginine and L-tryptophan were purchased from Dingguo Biotechnology Company (Beijing, China) and then each amino acid was dissolved in 0.85% saline (as a vehicle for a total injection volume of 5  μL) containing 0.1% Evans Blue solution to facilitate injection site localization. Based on similar/other experiments performed on chicks by other researchers [11,21,26], we selected two doses of each amino acid (0.15 or 1.5  μmol for L-leucine, 0.8 or 1.6  μmol for L-glutamate, 10 or 100  μg for L-tryptophan, and 20 or 200  μg for L-arginine).

### Intra-cerebroventricular (ICV) injection procedure

Chicks were injected using a micro-syringe using a method adapted from Davis et al. [27] and Cline et al. [28]. After data collection, the chick was decapitated and its head sectioned along the frontal plane to determine site of injection. Any chick without dye present in the lateral ventricle system was eliminated from analysis.

### Feed intake experiments

One hundred and forty four, 4-d old broiler chicks, fasted for 3 h, were randomly assigned (12 chicks per treatment) to receive L-leucine (0.15  μmol or 1.5  μmol in vehicle), L-glutamate (0.8 or 1.6  μmol in vehicle), L-tryptophan (10 or 100  μg in vehicle), L-arginine (20 or 200  μg) and vehicle control (0.85% saline containing 0.1% Evans Blue in the volume of 5 μL) by ICV administration. After injection, the chicks were returned to their individual cages and given *ad libitum* access to both feed and water. Feed intake was monitored at 0.25, 0.5, 1, 1.5 and 2 h post administration as follow. We gave chicks a certain amount of diet in a cup at the beginning of experiment (0 h) and weighed the remaining diet at various time points (0.25, 0.5, 1, 1.5 and 2 h), and then we could calculate the cumulative feed intake of different time points.

### RNA extraction

At 2 h after various amino acids ICV treatments, some chicks were slaughtered, and the hypothalamuses were quickly removed and snap frozen in liquid nitrogen. Total RNA was isolated from the hypothalamic tissue (about 50 mg) using TRIZOL reagent (Invitrogen, USA) and purified with DNase I (Invitrogen, USA) according to the manufacturer's instructions. The RNA concentration was determined using the photometer and the RNA had an average OD_260nm_: OD_280nm_ ratio between 1.8 and 2.0. The RNA quality was checked using 1.0% agarose gel electrophoresis.

### Reverse transcription and quantitative PCR

Synthesis of the first strand of cDNA was performed with N_10_ random primer and MMLV (promega, USA) using 4  μg of total RNA. In the first step, a mixture of 3 μL of the primer _(N10)_, 8 μL RNA and 4 μL of DEPC water was prepared in reaction tubes, and heated to 70°C for 5 m, then cooled with ice. In the second step, a mixture of 1 μL reverse transcriptase MMLV, 6 μL MMLV buffer, 0.5 μL RNase Inhibitor, 1.5 μL dNTPs, and 6 μL DEPC water was prepared. After overtaxing and a short centrifuge, this mixture was added to the reaction mixture in step one. The reaction mixture was then incubated at 37°C for 60 m, followed by 80°C for 5 m. This was properly stored at -30 for use in Real-time PCR.

Real-time PCR was performed using one-step SYBR Green PCR Mix (Takara, Dalian, China), containing MgCl_2_, dNTP, and Hotstar Taq polymerase. Primers were designed specifically for each gene by using Primers 5.0 software (PREMIER Biosoft International, USA). The reaction volumes and mixtures of reagents were as follows: 10 μL of 2× SYBR Green Master Mix, 0.5 μL primer (forward and reverse), 8.5 μL water, and 1 μL cDNA template. Amplification and melting curve analysis was performed in Stratergene Mx3005P (Agilent Technologies, USA). Melting curve analysis was conducted to confirm the specificity of each product and the sizes of the products verified on ethidium bromide-stained 1.0% agarose gels. The relative mRNA expression was calculated by 2^-ΔCt^ (ΔCt = Ct_target gene_-Ct_β-actin housekeeping gene_), and each gene expression in control group was presented as 100% [29]. Details of primer sequences, annealing temperatures, and lengths (bp) are presented in Table 1.

**Table 1:** Primer sequences and annealing temperatures

**Gene**	**Serial number**	**Primer sequences (5’-3’)**	**Annealing temp (°C)**	**Length (bp)**
β-actin	NM_205518	FP: CACCGCAAATGCTTCTAAAC	58	100
		RP: GCCATGCCAATCTCGTCTT		
NPY	NM_205473	FP: TGTTGAGGGAAAGCACAGAA	59	132
		RP: GATTTGCTTCAGAGGAGTGGA		
AgRP	AB489993	FP: CATCCTCACCTCGGACCTCA	63	111
		RP: GGGCCATCTGATCCAAGTCT		
POMC	NM_001031098	FP: AGAAGGGTTGGAACGAGA	61	99
		RP: TACACCTTGATGGGTCTCC		
MC4R	NM_001031514	FP: TGGAACCAGAGCAACGGAC	62	156
		RP: TGCCACAATGACCAAGACG		
CRF	AJ621492	FP: TCCCTGGACCTGACTTTCC	58	117
		RP: GCCTCACTTCCCGATGATTT		

### Statistical analysis

Data were analyzed by *t*-test (SPSS Statistics 17.0, SPSS Inc, USA); and values are represented as means ± S.E.M. Significance was established at the *P* < 0.05 level.

## Results

### ICV injection of leucine increased feed intake and hypothalamic NPY and AgRP mRNA expression

**Figure 1:**
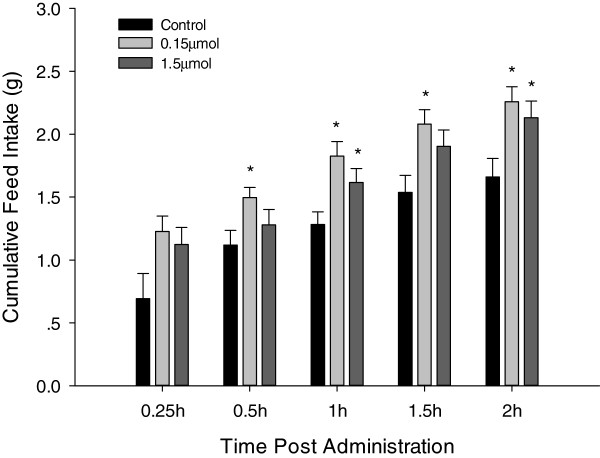
**Cumulative feed intake of broiler chicks after ICV administration with saline (control, n = 9), 0.15 μmol (n = 9) or 1.5 μmol (n = 9) L-leucine.** Feed intake was recorded at 0.25, 0.5, 1, 1.5, and 2 h post-administration; Data are represented as means ± S.E.M. Asterisk indicates significant difference from control group within each time point (*P* < 0.05).

To determine the effects of ICV injection of leucine on feed intake, 0.15  μmol and 1.5  μmol of leucine were administered. As shown in Figure 1, Feed intake was significantly (*P* < 0.05) increased at 0.5, 1, 1.5 and 2 h post-injection for the 0.15  μmol group, while for the 1.5  μmol group, there was a significant increase in feed intake when compared with control at 1 and 2 h post ICV injection; and at 1.5 h post-injection, feed intake was very close to a significant increase (*P* = 0.052). Feed intake was also increased at 0.5 h post-injection for the 1.5 μmol dose but was not statistically significant. Therefore, both concentrations of leucine produced a significant stimulatory effect on feed intake, with 0.15  μmol producing a relatively higher stimulatory effect up to 2 h post-injection.

**Figure 2:**
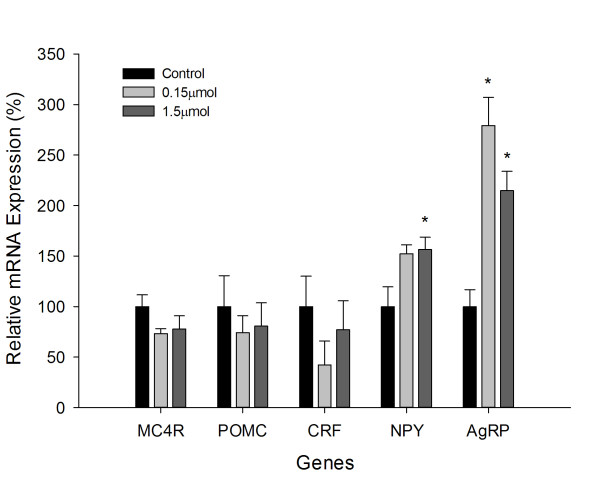
**Relative mRNA expression levels for hypothalamic MC4R, POMC, CRF, NPY and AgRP 2 h after ICV injection of leucine; Data are represented as means ± S.E.M.** (n = 6). Asterisk indicates significant difference from control group within each time point (*P* < 0.05).

ICV injection of leucine significantly increased both AgRP and NPY mRNA expressions in the hypothalamus (Figure 2). Both doses of leucine significantly increased the mRNA expression levels of AgRP, but only high dose (1.5  μmol) significantly increased the mRNA expression levels of NPY. NPY mRNA was however increased to almost significant levels for the 0.15  μmol (*P* = 0.058). The anorexigenic Neuropeptide POMC and CRF, and the melanocortin receptor MC4R, had their mRNA expression levels reduced though the reduction did not reach statistically significant levels for both doses of leucine used.

### Glutamate ICV injection reduced feed intake but increased hypothalamic MC4R and CRF mRNA expression

**Figure 3:**
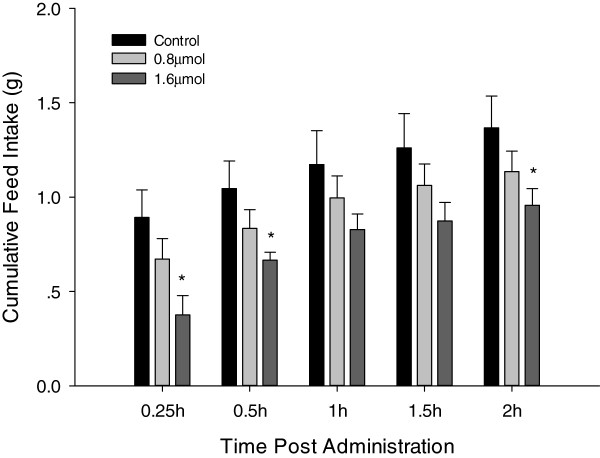
**Cumulative feed intake of broiler chicks after ICV administration with saline (control, n = 9), 0.8 μmol (n = 10) or 1.6 μmol (n = 7) L-glutamate.** Feed intake was recorded at 0.25, 0.5, 1, 1.5, and 2 h post-administration; Data are represented as means ± S.E.M. Asterisk indicates significant difference from control group within each time point (*P* < 0.05).

To determine the effects of Glutamate ICV injection on feed intake, 0.8  μmol and 1.6  μmol of L-glutamate were administered (Figure 3). Feed intake was significantly (*P* < 0.05) decreased at 0.25, 0.5 and 2 h post-injection when a dose of 1.6  μmol was administered. For the same dose, feed intake at 1, and 1.5 h post-injection reduced despite not reaching statistically significant levels. In a similar manner, feed intake was reduced when a dose of 0.8  μmol was administered but not significantly. Therefore, glutamate elicited a dose dependant inhibitory effect on feed intake in broiler chicks, with a higher dose significantly decreasing feed intake.

**Figure 4:**
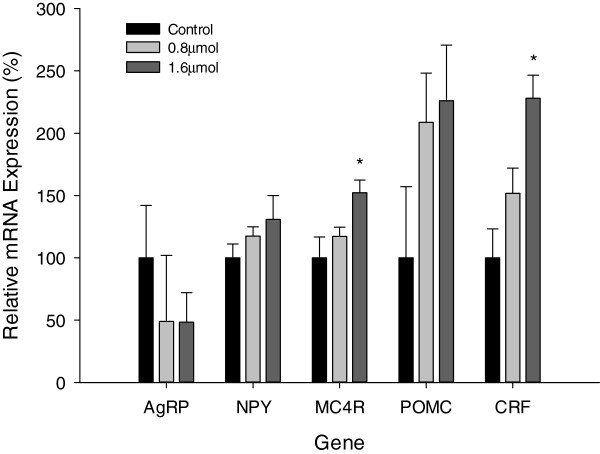
**Relative mRNA expression levels for hypothalamic AgRP, NPY, MC4R, POMC and CRF 2 h after glutamate ICV injection; Data are represented as means ± S.E.M.** (n = 6). Asterisk indicates significant difference from control group within each time point (*P* < 0.05).

The MC4R mRNA expression level was significantly (*P* = 0.02) increased after glutamate ICV injection with a 1.6  μmol concentration (Figure 4). For the lower ICV injection concentration of 0.8  μmol, there was no significant increase in the mRNA expression level of MC4R in the hypothalamus. CRF mRNA expression level was also significantly increased (*P* = 0.015) for the higher ICV injection concentration of 1.6  μmol but not for the lower concentration of 0.8 μmol. However, POMC mRNA expression levels at both concentrations of glutamate ICV injection showed an insignificant increase. AgRP mRNA expression level was reduced but not significantly, while NPY mRNA expression level did not significantly increase. The results clearly show an up-regulation of the anorexigenic factors though there were no significant changes in mRNA expression levels of orexigenic factors.

### Tryptophan ICV injection had no significant effect on food intake in broiler chicks

**Figure 5:**
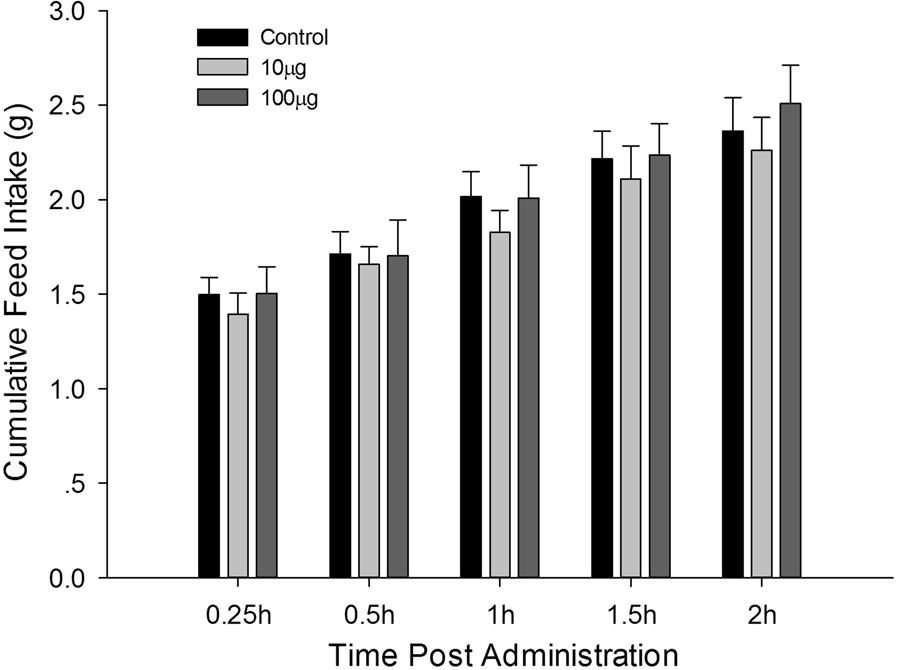
**Cumulative feed intake of broiler chicks after ICV administration with saline (control, n = 9), 10 μg (n = 8) or 100 μg (n = 9) L-tryptophan.** Feed intake was recorded at 0.25, 0.5, 1, 1.5, and 2 h post-administration; Data are represented as means ± S.E.M.

10 and 100  μg of L-tryptophan were administered in order to determine the effects of tryptophan ICV injection on feed intake in broiler chicks. There was clearly no significant difference comparing with the control in feed intake when a higher concentration (100  μg) was used. However, the 10  μg L-tryptophan dose minimally reduced feed intake throughout post-injection period (2 h): 0.25 h (*P* = 0.189), 1 h (*P* = 0.224), and 1.5 h post-injection (*P* = 0.270) when compared with control. Although not significant, the minimal feed intake reduction was uniform and consistent. A summary of the results is presented in Figure 5.

### Arginine ICV injection did not significantly affect feed intake

**Figure 6:**
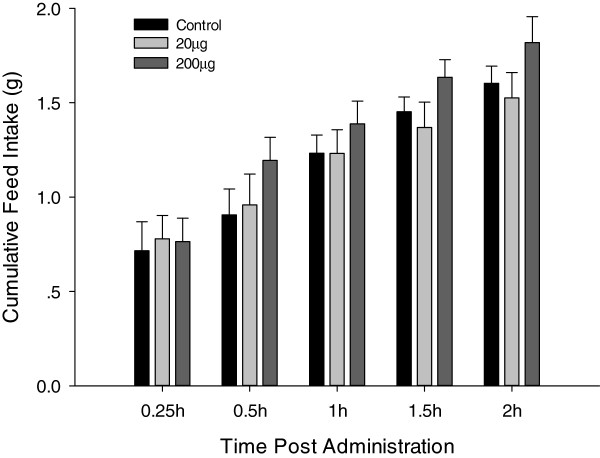
Cumulative Feed Intake of broiler chicks after ICV administration with saline (control, n = 8), 20 μg (n = 9) or 200 μg (n = 7) L-arginine, feed intake was recorded at 0.25, 0.5, 1, 1.5, and 2 h post-administration; Data are represented as means ± S.E.M.

As shown in Figure 6, ICV injection of L-arginine (20 and 200  μg) did not significantly affect feed intake, despite of the higher dose (200  μg) showing a steady minimal increase in feed intake comparing with the control from 0.5 h post-injection (*P*-values = 0.192, 0.269 and 0.245 at 0.5, 1.5 and 2 h post-injection, respectively).

## Discussion

The main conclusion to be drawn from the present results was that ICV injection of leucine stimulates feed intake in broiler chicks. For the lower dose (0.15  μmol) of leucine, the increase of feed intake was significant from 0.5 h post-injection and this increase was sustained up to 2 h post-injection. The higher dose of leucine stimulated a significant increase in feed intake from 1 h post-injection, sustaining it up to 2 h post-injection. It was therefore the lower dose that was more effective at influencing feed intake. Moreover, ICV injection of leucine significantly increased the mRNA expression levels of the orexigenic Neuropeptide NPY and AgRP.

NPY is a potent hypothalamic orexigenic peptide. Central administration or over-expression of NPY resulted in significant increases of food intake and body weight [30,31]. In contrast, induced selective ablation or knockdown of NPY or AgRP neurons in adult mice led to reduction of feed intake [32,33]. AgRP, an antagonist of *α*-MSH in chickens, can exert its orexigenic effects through binding to specific melanocortin receptor subtypes (MC3-R and MC4-R) [34]. In our study, the mRNA expression levels of hypothalamic orexigenic Neuropeptide NPY were significantly increased by ICV injection of leucine. And this suggested that leucine was able to trigger increased genetic transcription of orexigenic Neuropeptide (NPY/AgRP) within hypothalamic neurons, thereby causing an increase in feed intake.

The findings of this study were consistent with a research performed on leghorn chicks [11], where ICV injection of 200 μg leucine stimulated feeding behavior. Izumi et al (2004) proposed that a metabolite of leucine increases feed intake in chicks. This was because leucine was transaminase to produce glutamate and α-ketoisocaproic acid, and α-ketoisocaproic acid was converted to acetoacetyl-CoA in the brain [11,35]. However, ICV injection of α-ketoisocaproate had no effect on food intake, suggesting that it was not this metabolite of leucine increased food intake in chicks [11]. Thus, it was assumed that glutamate resulting from the exogenous leucine stimulated feeding behavior. However, the present study has determined that glutamate ICV injection inhibited feed intake in the broiler chicks. Therefore, it was still unclear whether a metabolite of leucine increases feed intake or not, and if not, how leucine was able to modulate this feeding effect in broiler chicks is yet to be elucidated. But it was still likely that leucine or its metabolite modulate its effects through the activation of AMPK-dependent mechanisms leading to the inhibition of mTOR activity and therefore a stimulation of feeding behavior in broiler chicks. Further experiments to investigate signaling pathways of leucine mediating effects on feeding behavior in poultry were indeed required.

The present study has also determined that ICV injection of glutamate inhibits food intake in broiler chicks. Significant increases in the mRNA levels of MC4R and CRF were also observed 2 h post-injection. These results suggested that L-glutamate acted within the hypothalamus to inhibit food intake, and might exert its effects in collaboration with the anorexigenic genes, including CRF, MC4R and POMC.

Although not significantly, in this study, POMC gene mRNA levels were up-regulated by ICV injection of glutamate, and this was in line with research findings that a proportion of POMC/CART neurons were glutamatergic since they had been reported to contain the vesicular glutamate transporter 2 (VGLUT2), a marker for glutamatergic neurons [36]. Endogenous POMC neurons exerted a tonic inhibitory effect on feeding and energy storage via their release of desacetyl-α-MSH, the primary melanocortin cleavage product in the brain, at downstream sites containing MC4-R [37]. Both NPY and AgRP orexigenic Neuropeptide did not show a significant change in mRNA expression levels, consistent with other previous research results [38] that these neurons were not glutamatergic but GABAergic.

Baghbanzadeh and Babapour (2007) suggested that glutamate, acting as a neurotransmitter, is involved in feed intake regulation in broiler cockerels, and that this effect was probably mediated by both ionotropic and metabotropic receptors [15]. The results of this study were also consistent with the research result that ICV injection of glutamate in pigeons was able to decrease feed intake, in a study to explore the possible involvement of glutamatergic mechanisms in the control of food intake [12]. On the other hand, inhibition of vesicular glutamate uptake could increase feed intake in broilers [39]. ICV treatments with N-methyl-D-aspartic acid (NMDA) or D, L-a-amino-3-hydroxy-isoxazole prop ionic acid (AMPA) decreased feed intake in 24 h-food deprived pigeons. In free-feeding pigeons, glutamatergic receptor antagonists MK-801 and CNQX treatments significantly increased both food intake and feeding duration [12]. Therefore, combination of the above results and the findings in the present study implied that glutamate-mediated circuits, mediated by AMPA and NMDA receptors, played a role in significantly inhibiting feed intake in broiler chicks.

The present findings contrasted with those obtained in mammals. Systemic, ICV or local injections of glutamate or its agonists into the lateral hypothalamus elicits a dose-dependent stimulation of feed intake in mammals [14,15,40]; whereas tuberal lateral hypothalamic injection of an NMDA antagonist suppressed feeding elicited by NMDA [38,41]. The fact that glutamate stimulated food intake after injection in the lateral hypothalamus in mammals could be explained by an excitatory role of glutamate on orexigenic MCH and orexin-containing neurons [38].

The results of this study showed that tryptophan ICV injection did not significantly affect food intake in 3 h-fasted broiler chicks. This was despite the 10  μg dose leading to a steady minimal decrease in feed intake throughout the 2 h -post-injection period. It might be that the 10  μg of L-tryptophan was too low whereas the 100  μg dose was too high to significantly affect feed intake. In fact, a study by Bungo et al (2008) showed that L-tryptophan ICV injection of 3d old chicks fed *ad libitum* significantly suppressed feed intake, suggesting that tryptophan injected into the brain of chicks was promptly converted to serotonin (5-hydroxytryptamine 5-HT) and induced hypophagia via the 5-HT2A receptors [21]. There was thus still need to further investigate the effects of central tryptophan on food intake in broiler chicks under different physiological conditions.

L-arginine ICV injection failed to significantly affect feed intake in broiler chicks, though a higher injection dose (200  μg) showed a steady minimal and statistically not significant increase in feed intake from 0.5 h post-injection. These results were consistent with those of Calapai et al (1998) where a 10  μg L-arginine ICV injection dose did not affect feed intake in mice. Moreover Calapai et al (1998) showed that L-arginine (administered together with leptin) antagonized the leptin-induced food intake reduction in mice, and linked this effect to the NO pathway [23]. In addition, supplementation of canola meal-based diets with arginine significantly increased feed intake in broilers [42]. Our results therefore suggested that arginine alone might not centrally and directly affect feed intake in broiler chicks.

In conclusion, the results presented in this study suggested that L-leucine and L-glutamate could act within the hypothalamus to influence food intake, and both orexigenic and anorexigenic Neuropeptide might contribute directly to this effect. ICV injection of leucine increased feed intake and hypothalamic NPY and AgRP mRNA expression, while glutamate ICV injection reduced feed intake but increased hypothalamic MC4R, CRF and POMC mRNA expression levels. Tryptophan and arginine may not directly affect feed intake when ICV injected in broiler chicks.

## Competing interests

The authors declare that they have no competing interests.

## Authors' contributions

QYJ, SBW and PK conceived the experiment and drafted the manuscript. SBW charted the figures, preformed the statistical analysis and revised the manuscript. PK carried the feed intake experiment. SFC and JJY participated in the gene expression experiment. GS, XTZ, LW, PG, QYX and YLZ participated in the design of the study. All authors have read and approved the final manuscript.

## References

[B1] Morrison CD Xi X White CL Ye J Martin RJ Amino acids inhibit Agrp gene expression via an mTOR-dependent mechanism Am J Physiol Endocrinol Metab 2007 293 E165 E171 10.1152/ajpendo.00675.2006 17374702 PMC2596875

[B2] Ribeiro EB Studying the central control of food intake and obesity in rats Revista De Nutricao-Brazilian Journal of Nutrition 2009 22 163 171

[B3] Lenard NR Berthoud HR Central and peripheral regulation of food intake and physical activity: pathways and genes Obesity 2008 16 S11 S22 19190620 10.1038/oby.2008.511 PMC2687326

[B4] Schwartz MW Woods SC Porte D Jr Seeley RJ Baskin DG Central nervous system control of food intake Nature 2000 404 661 671 10766253 10.1038/35007534

[B5] Cota D Proulx K Smith KA Kozma SC Thomas G Woods SC Seeley RJ Hypothalamic mTOR signaling regulates food intake Science 2006 312 927 930 10.1126/science.1124147 16690869

[B6] Denbow DM Food intake control in birds Neurosci Biobehav Rev 1985 9 223 232 10.1016/0149-7634(85)90047-8 3892379

[B7] Denbow DM Food intake regulation in birds J Exp Zool 1999 283 333 338 10.1002/(SICI)1097-010X(19990301/01)283:4/5<333::AID-JEZ3>3.0.CO;2-R

[B8] Blouet C Jo YH Li X Schwartz GJ Mediobasal hypothalamic leucine sensing regulates food intake through activation of a hypothalamus-brainstem circuit J Neurosci 2009 29 8302 8311 10.1523/JNEUROSCI.1668-09.2009 19571121 PMC2740923

[B9] Proud CG mTOR-mediated regulation of translation factors by amino acids Biochem Biophys Res Commun 2004 313 429 436 10.1016/j.bbrc.2003.07.015 14684180

[B10] Gloaguen M Le Floc'h N Corrent E Primot Y van Milgen J Providing a diet deficient in valine but with excess leucine results in a rapid decrease in feed intake and modifies the postprandial plasma amino acid and alpha-keto acid concentrations in pigs J Anim Sci 2012 [Published online before print May 14, 2012] 10.2527/jas.2011-4956 22585822

[B11] Izumi T Kawamura K Ueda H Bungo T Central administration of leucine, but not isoleucine and valine, stimulates feeding behavior in neonatal chicks Neurosci Lett 2004 354 166 168 10.1016/j.neulet.2003.09.071 14698464

[B12] Zeni LAZR Seidler HBK De Carvalho NAS Freitas CG Marino-Neto J Paschoalini MA Glutamatergic control of food intake in pigeons: effects of central injections of glutamate, NMDA, and AMPA receptor agonists and antagonists Pharmacol Biochem Behav 2000 65 67 74 10.1016/S0091-3057(99)00153-7 10638638

[B13] Bisaga A Danysz W Foltin RW Antagonism of glutamatergic NMDA and mGluR5 receptors decreases consumption of food in baboon model of binge-eating disorder Eur Neuropsychopharmacol 2008 18 794 802 10.1016/j.euroneuro.2008.05.004 18573641 PMC2591926

[B14] Reddy VM Meharg SS Ritter S Dose-related stimulation of feeding by systemic injections of monosodium glutamate Physiol Behav 1986 38 465 469 10.1016/0031-9384(86)90412-9 3823160

[B15] Baghbanzadeh A Babapour V Glutamate ionotropic and metabotropic receptors affect feed intake in broiler cockerels J Vet Res 2007 62 125 129

[B16] Zendehdel M Baghbanzadeh A Babapour V Cheraghi J The effects of bicuculline and muscimol on glutamate-induced feeding behavior in broiler cockerels J Comp Physiol A Neuroethol Sens Neural Behav Physiol 2009 195 715 720 10.1007/s00359-009-0446-3 19415297

[B17] Taati M Nayebzadeh H Zendehdel M The effects of DL-AP5 and glutamate on ghrelin-induced feeding behavior in 3-h food-deprived broiler cockerels J Physiol Biochem 2011 67 217 223 10.1007/s13105-010-0066-y 21203879

[B18] Eder K Peganova S Kluge H Studies on the tryptophan requirement of piglets Arch Tierernahr 2001 55 281 297 10.1080/17450390109386198 12357590

[B19] Le Floc'h N Seve B Biological roles of tryptophan and its metabolism: Potential implications for pig feeding Livest Sci 2007 112 23 32 10.1016/j.livsci.2007.07.002

[B20] Emadi M Kaveh K Jahanshiri F Hair-Bejo M Ideris A Alimon AR Dietary tryptophan effects on growth performance and blood parameters in broiler chicks J Anim Vet Adv 2010 9 700 704

[B21] Bungo T Yahata K Izumi T Dodo KI Yanagita K Shiraishi JI Ohta Y Fujita M Centrally administered tryptophan suppresses food intake in free fed chicks through the serotonergic system J Poult Sci 2008 45 215 219 10.2141/jpsa.45.215

[B22] Morris SM Jr Enzymes of arginine metabolism J Nutr 2004 134 2743S 2747S discussion 2765S-2767S 15465778 10.1093/jn/134.10.2743S

[B23] Calapai G Corica F Allegra A Corsonello A Sautebin L De Gregorio T Di Rosa M Costantino G Buemi M Caputi AP Effects of intracerebroventricular leptin administration on food intake, body weight gain and diencephalic nitric oxide synthase activity in the mouse Br J Pharmacol 1998 125 798 802 10.1038/sj.bjp.0702121 9831917 PMC1571026

[B24] Gaskin FS Farr SA Banks WA Kumar VB Morley JE Ghrelin-induced feeding is dependent on nitric oxide Peptides 2003 24 913 918 10.1016/S0196-9781(03)00160-8 12948844

[B25] Yang SJ Denbow DM Interaction of leptin and nitric oxide on food intake in broilers and leghorns Physiol Behav 2007 92 651 657 10.1016/j.physbeh.2007.05.009 17631366

[B26] Suenaga R Tomonaga S Yamane H Kurauchi I Tsuneyoshi Y Sato H Denbow DM Furuse M Intracerebroventricular injection of L-arginine induces sedative and hypnotic effects under an acute stress in neonatal chicks Amino Acids 2008 35 139 146 10.1007/s00726-007-0610-4 18163184

[B27] Davis JL Masuoka DT Gerbrandt LK Cherkin A Autoradiographic distribution of L-proline in chicks after intracerebral injection Physiol Behav 1979 22 693 695 10.1016/0031-9384(79)90233-6 482410

[B28] Cline MA Nandar W Smith ML Pittman BH Kelly M Rogers JO Amylin causes anorexigenic effects via the hypothalamus and brain stem in chicks Regul Pept 2008 146 140 146 10.1016/j.regpep.2007.09.003 17916389

[B29] Livak KJ Schmittgen TD Analysis of relative gene expression data using real-time quantitative PCR and the 2(-Delta Delta C(T)) method Methods 2001 25 402 408 10.1006/meth.2001.1262 11846609

[B30] Stanley BG Leibowitz SF Neuropeptide Y: stimulation of feeding and drinking by injection into the paraventricular nucleus Life Sci 1984 35 2635 2642 10.1016/0024-3205(84)90032-8 6549039

[B31] Nguyen AD Herzog H Sainsbury A Neuropeptide Y and peptide YY: important regulators of energy metabolism Curr Opin Endocrinol Diabetes Obes 2011 18 56 60 10.1097/MED.0b013e3283422f0a 21157324

[B32] Luquet S Perez FA Hnasko TS Palmiter RD NPY/AgRP neurons are essential for feeding in adult mice but can be ablated in neonates Science 2005 310 683 685 10.1126/science.1115524 16254186

[B33] Gardiner JV Kong WM Ward H Murphy KG Dhillo WS Bloom SR AAV mediated expression of anti-sense neuropeptide Y cRNA in the arcuate nucleus of rats results in decreased weight gain and food intake Biochem Biophys Res Commun 2005 327 1088 1093 10.1016/j.bbrc.2004.12.113 15652508

[B34] Richards MP Genetic regulation of feed intake and energy balance in poultry Poult Sci 2003 82 907 916 12817445 10.1093/ps/82.6.907

[B35] Yudkoff M Brain metabolism of branched-chain amino acids Glia 1997 21 92 98 10.1002/(SICI)1098-1136(199709)21:1<92::AID-GLIA10>3.0.CO;2-W 9298851

[B36] Meister B Neurotransmitters in key neurons of the hypothalamus that regulate feeding behavior and body weight Physiol Behav 2007 92 263 271 10.1016/j.physbeh.2007.05.021 17586536

[B37] Cone RD The central melanocortin system and energy homeostasis Trends Endocrinol Metab 1999 10 211 216 10.1016/S1043-2760(99)00153-8 10407394

[B38] Collin M Backberg M Ovesjo ML Fisone G Edwards RH Fujiyama F Meister B Plasma membrane and vesicular glutamate transporter mRNAs/proteins in hypothalamic neurons that regulate body weight Eur J Neurosci 2003 18 1265 1278 10.1046/j.1460-9568.2003.02840.x 12956725

[B39] Baghbanzadeh A Modirsaneie M Emam G Hajinezhad M Microhandling of vesicular glutamate uptake modulate feeding in broilers J Anim Physiol Anim Nutr (Berl) 2010 94 74 77 10.1111/j.1439-0396.2008.00887.x 19175460

[B40] Stricker-Krongrad A Beck B Nicolas JP Burlet C Central effects of monosodium glutamate on feeding behavior in adult Long-Evans rats Pharmacol Biochem Behav 1992 43 881 886 10.1016/0091-3057(92)90421-B 1448482

[B41] Stanley BG Willett VL 3rd Donias HW Dee MG 2nd Duva MA Lateral hypothalamic NMDA receptors and glutamate as physiological mediators of eating and weight control Am J Physiol 1996 270 R443 449 8779877 10.1152/ajpregu.1996.270.2.R443

[B42] Khajali F Tahmasebi M Hassanpour H Akbari MR Qujeq D Wideman RF Effects of supplementation of canola meal-based diets with arginine on performance, plasma nitric oxide, and carcass characteristics of broiler chickens grown at high altitude Poult Sci 2011 90 2287 2294 10.3382/ps.2011-01618 21934012

